# Ultra-high resolution imaging of laminar thickness in face-selective cortex in autism

**DOI:** 10.3758/s13415-025-01298-w

**Published:** 2025-04-30

**Authors:** Rankin W. McGugin, Allen T. Newton, Brianna J. Lewis, Caitlin A. Convery, Ekomobong E. Eyoh, Isabel Gauthier, Carissa J. Cascio

**Affiliations:** 1 Department of Psychology, Vanderbilt University, 111 21 st Avenue South, Nashville, TN 37240 USA; 2 Department of Radiology and Radiological Sciences, Vanderbilt University Medical Center, Nashville, TN USA; 3 Department of Psychiatry and Behavioral Sciences, Vanderbilt University Medical Center, Nashville, TN USA; 4 Vanderbilt Brain Institute, Vanderbilt University, Nashville, TN USA; 5 Frist Center for Autism and Innovation, Vanderbilt University, Nashville, TN USA; 6 Vanderbilt Kennedy Center, Vanderbilt University Medical Center, Nashville, TN USA; 7 Vanderbilt University Institute of Imaging Science, Vanderbilt University, Nashville, TN USA; 8 Institute of Child Development, University of Minnesota, Minneapolis, MN USA

**Keywords:** Individual differences, Autism, Face recognition, MRI

## Abstract

Gray matter cortical thickness (CT) is related to perceptual abilities. The fusiform face area (FFA) (Kanwisher et al., *The Journal of Neuroscience: The Official Journal of the Society for Neuroscience*, *17*, 4302–4311, 1997) in the inferior temporal lobe is defined by its face selectivity, and the CT of the FFA correlates with the ability to make difficult visual decisions (Bi et al., *Current Biology*, *24*, 222–227, 2014; McGugin et al., *Journal of Cognitive Neuroscience*, *28*, 282–294, 2016, *Journal of Cognitive Neuroscience*, *32*, 1316–1329, 2020). In McGugin et al. *Journal of Cognitive Neuroscience*, *32*, 1316–1329, (2020), individuals with better face recognition had relatively thinner FFAs, whereas those with better car recognition had thicker FFAs. This opposite correlation effect (OCE) for faces and cars was pronounced when we look selectively at the deepest laminar subdivision of the FFA. The OCE is thought to arise because car and face recognition abilities are fine-tuned by experience during different developmental periods. Given autism’s impact on face recognition development, we predicted the OCE would not appear in autistic individuals. Our results replicate the OCE in total FFA thickness and in deep layers in neurotypical adults. Importantly, we find a significant reduction of these effects in adults with autism. This supports the idea that the OCE observed in neurotypical adults has a developmental basis. The abnormal OCE in autism is specific to the right FFA, suggesting that group differences depend on local specialization of the FFA, which did not occur in autistic individuals.

**Supplementary information:**

The online version contains supplementary material available at 10.3758/s13415-025-01298-w.

## Introduction

Cortical thickness (CT) is related to perceptual abilities. One fascinating example is the relationship between the structure of the fusiform face area (FFA) (Kanwisher et al., 1997) and face and object recognition abilities. The FFA is a functional region in the inferior temporal cortex that is defined by its face selectivity. Most research on the FFA and its relationship to behavior focuses on functional, rather than structural properties of the cortex. Nonetheless, those with better face recognition have relatively thinner FFAs (Bi et al., 2014; McGugin et al., 2016). Surprisingly, in the same region and the same individuals, we find the opposite pattern for the recognition of cars (and other vehicles)—those with better car recognition have a relatively thicker FFA (McGugin et al., 2016). The striking difference between the effect for faces and cars could be due to people acquiring experience with faces much earlier than with cars. According to this explanation, the two abilities would interact with different neurodevelopmental processes at different times in brain development. We replicated this cross-over interaction in 14 adult men and used ultra-high resolution imaging to investigate these relationships at a microlevel, in the laminar structure of the FFA (McGugin et al., 2020). Men with better face recognition had relatively thinner FFAs, and this effect was specific to the deepest cortical layers of the FFA. Those with better vehicle recognition had relatively thicker FFAs, and this was represented generally across all cortical layers. Here, we aim to replicate the cross-over interaction in total cortical thickness and specifically in the deep layers of this area and test a prediction that it would differ in autistic individuals. Before we discuss our predictions, we first explain what is special about faces and cars and how face recognition is expected to differ in autistic individuals.

The choice of car recognition to compare with face recognition in this line of research has many precedents, and it has come with some surprises. The ability to discriminate car models was first measured in a study that followed up on work showing that training with novel objects led to increased selectivity for these objects in the FFA. People varying in car and bird expertise also engaged the FFA (Gauthier et al., 2000; see also Xu, 2005), and a meta-analysis has found that such functional effects of expertise in FFA are robust (Burns et al., 2019). Experience with cars seems to recruit FFA representations just like experience with other objects would, and in the same direction. In that spirit, other authors have used cars as a proxy for general object recognition. There are, however, good reasons to doubt that cars are representative of most objects. In large individual differences studies, cars and faces have been the two categories found least correlated with performance for other objects and also minimally correlated with one another (McGugin et al., 2012). One suggestion is that we have more experience individuating cars and faces than most other objects (Sunday et al., 2019). Even in this context, the finding that the FFA CT is strongly correlated to both face and car recognition, but in opposite directions (McGugin et al., 2020), would not have been predicted from what was known about faces and cars. McGugin et al. (2020) proposed that distinct mechanisms influenced CT for the two categories, because car and face recognition abilities are fine-tuned by experience at different periods in development.

While a cross-sectional approach cannot measure developmental processes directly, an explanation rooted in experience-dependent plasticity that occurs at different stages in brain development for faces and cars leads to predictions that can be tested. We investigate some of these predictions in individuals with a developmental disorder that impacts face recognition. Autism is an early-onset, lifespan-persistent neurodevelopmental disorder characterized by social communication deficits and repetitive patterns of behavior. The highly prevalent (Dellapiazza et al., 2020) sensory and perceptual differences in autism are hypothesized to cascade to influence social function (Baranek et al., 2017; Hilton et al., 2010; Thye et al., 2018). Decoding of faces is a foundational perceptual skill that serves as a precursor to more complex social abilities, and substantial deficits in face processing are reported in autism (Griffin et al., 2021). Automatic orienting to faces is intact (de Klerk et al., 2014; Guillon et al., 2014), but later-developing processes (i.e., decoding (Key & Stone, 2012; McPartland et al., 2011), identifying (Weigelt et al., 2012), and interpreting (Deutsch & Raffaele, 2019; Lozier et al., 2014) faces) are affected throughout the lifespan. Importantly, the altered trajectory of face processing ability begins in infancy for autistic individuals (Chawarska & Volkmar, 2007; Shic et al., 2014) and persists into adulthood (Kamensek et al., 2024). Delayed development of face processing (Chawarska & Volkmar, 2007; de Klerk et al., 2014; Shephard et al., 2020; Tye et al., 2022), altered attention to faces (Macari et al., 2021; Shic et al., 2014), and high interest in machines (Turner-Brown et al., 2011; Uljarević et al., 2022) could result in face and car recognition abilities beginning at more similar times for those with autism than they do in typical development.

The neural basis of these face effects in autism have been reported in multiple large-scale studies finding group differences in CT of the fusiform gyrus (Libero et al. 2014; Zoltowski et al. 2021; Cárdenas-de-la-Parra et al. 2021), but these studies have not functionally localized the FFA and did not address behavioral abilities. In addition, a recent large sample study (Chen et al., 2023) with 1,053 participants from the Human Connectome Project (Van Essen et al., 2013) supported the existence of two distinct face selective regions in the FG: mFus-faces/FFA2 is more anterior and pFusfaces/FFA1 more posterior (Weiner et al., 2014a, 2018). The two areas differ in their functional responses and architectural and connectivity characteristics. Our current methods for laminar measurement require us to focus on a single region of interest, and FFA2 has been more specifically linked with functional effects of expertise (Golarai et al., 2007; McGugin et al., 2014, 2015, 2018) and is where the opposite effects for cars and faces was previous reported (McGugin et al., 2020).

In the current work, the pattern of interest is this crossover interaction between car and face recognition with their relationship to CT. In McGugin et al. (2020), the two opposite correlations were essentially statistically independent effects—consistent with the idea that they reflect influences that occur at different times. This independence allows us to simplify our predictions and increase our statistical power by directly testing the opposite correlations for car and face correlations with CT (i.e., the opposite correlation effect [OCE]) in autism and controls. A high OCE indicates that the correlations are strong and in opposite directions, whereas a low OCE indicates that the correlations are not very different from one another. We focus our analyses where the crossover effect was strongest in McGugin et al. (2020)—total CT and deep layers—and thereby ground our confirmatory analyses into a commonsense approach to statistical testing. In this work, we will test the following hypotheses: 1) Face recognition will be selectively impaired in people with autism; 2) We expect a significant OCE in total CT as well as deep layers for controls; 3) We expect a reduced OCE in total CT as well as deep layers, relative to controls, in adults with autism; 4) The abnormal OCE will be specific to the FFA and not a general property of the FG more broadly.

## Methods

### Power analysis

We calculated the OCE in the results of McGugin et al. (2020). We multiplied standardized face recognition ability scores and added them to standardized car recognition ability scores. This index was then correlated with CT measures. The resulting OCE was 0.946 for total CT, 0.506 for superficial layers CT, 0.466 for middle layers CT, and 0.752 for deep layers CT. To calculate *a priori* power of finding a reduction of this effect in people with autism, we assumed a much smaller OCE (although, to be conservative, perhaps not quite null) of 0.1 in the autism group. We then used the R package “cower” (https://rdrr.io/github/m-Py/cower/f/README.md) to calculate the sample size per group required to find a difference between OCEs for the two groups, with 80% power. In total CT, superficial, middle, and deep layers CT, respectively, that sample size is 9, 77, 97, and 24. This illustrates how sharply power depends on effect size. In other words, given our current sample sizes (16 vs. 17), we reach the following levels of power for these comparisons: 99%, 22%, 19%, and 62%. We therefore abandoned the idea of testing differences between groups in middle and superficial layers and focused on total CT and deep layers.

### Participants, clinical characterization, and measurement

We collected data from 38 participants. Five were excluded: three owing to excessive motion during the MRI, one owing to hardware malfunction, and one owing to a failure to confirm autism diagnosis. Our final sample included 16 autistic adults (AUT) [male/female: 9/7; age: mean = 26.6, range = 18–40] and 17 adults with typical developmental histories (TD) [male/female: 6/11; age: mean = 27.5, range = 18–48] matched in age, biological sex, handedness, and education. The sample was recruited from the following pools of potential participants: previous studies in the Cascio lab; Vanderbilt Psychiatric Hospital; Vanderbilt Treatment and Research Institute for Autism Spectrum Disorders; Autism Tennessee; Nashville area clinics; and local community message boards. The following general inclusion criteria applied to all participants: 1) age between 18 and 55 years; 2) no diagnosed organic brain disease, brain lesions, history of head traumas, or neurological disorders; 3) no substance/alcohol abuse/dependence during the past 2 years; 4) normal or corrected-to-normal hearing and vision; and 5) IQ > 70, assessed with the Wechsler Abbreviated Scales of Intelligence (Wechsler, 2011). Self-reported adaptive and maladaptive functioning, indexing risk for psychiatric or psychological conditions, was assessed with the Achenbach System of Empirically Based Assessment Adult Form (Achenbach & Rescorla, 2010). Magnetic resonance imaging (MRI) contraindications, including claustrophobia, nonremovable ferrous metal in the body, or pacemakers, served as exclusion criteria. All adults were given materials to prepare for scanning appointments, including a “what to expect” document, scanner sounds to play at home, and practice instructions. Adults with no previous MRI history, or with any anxiety about the MRI environment, were scheduled for a mock scan session in the Vanderbilt University Institute of Imaging Science (VUIIS) mock scanning suite. The mock scanner is a nonworking MRI shell that simulates the experience of the MRI.

Adults with autism received diagnostic confirmation using Module 4 of the ADOS- 2 (Lord et al., 2012), which was administered by a research-reliable clinical psychologist with experience in diagnosing autism. Only participants with an ADOS score of 7 or higher on the social + communication algorithm and clinical judgment were included in the autism group. Live clinical impressions and ADOS algorithms were supplemented with a parent interview based on the algorithm items of the ADI-R (Lord et al., 1994) when a parent was available. However, because the reliability of the ADI-R drops for adults without intellectual disability (Fusar-Poli et al., 2017) and parents were not universally available, this was not used as an inclusion criterion.

### Behavioral testing

We used a battery of six visual tasks, including a memory- and matching-based task each for three categories: faces, cars, and novel objects (used as an additional control category). The tasks were completed in the same order for all participants to avoid confounding order effects with individual differences.

First, participants completed the extended version of the Cambridge Face Memory Test (CFMT+ (Duchaine & Nakayama, 2006; Russell et al., 2009). In the CFMT+, participants first studied frontal views of unfamiliar male faces, followed by introductory learning trials. Participants were then given forced-choice test displays containing one target face and two distractor faces, where they were instructed to select the face that matched one of the original target faces. In four sections of the test, matching faces varied from their original presentation in their lighting, pose, and/or degree of visual noise. For a complete description of the CFMT+, see Russell et al. (2009).

Second, participants completed the Cambridge Car Memory Test (CCMT), matched and modeled after the CFMT. For a complete description of the CCMT, see Dennett et al. (2012).

**Fig. 1:**
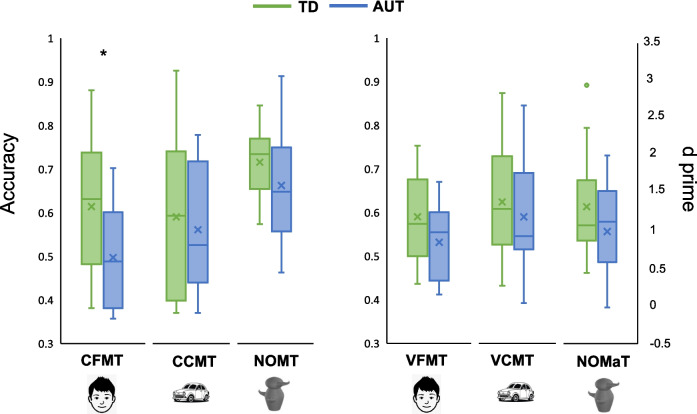
Behavioral performance across object categories and groups. Behavioral performance on memory tests (left; Cambridge Face Memory Test (CFMT), Cambridge Car Memory Test (CCMT), Novel Object Memory Test (NOMT)) and matching tests (right; Vanderbilt Face Matching Test (VFMT), Vanderbilt Car Matching Test (VCMT), Novel Object Matching Test (NOMaT)) for typical development (TD) and autism (AUT) groups. The NOMaT is scored as d prime; all other tests are scored by accuracy. Box plots show the range and mean of behavioral performance within and across groups. Asterisks denote significant group differences (*p* <.05 with Bonferroni correction for multiple comparisons)

Third, participants completed the Novel Object Memory Test (NOMT; Richler et al., 2019) where they first study six exemplars from a given category for as long as they liked. They then completed two 24-trial blocks (48 trials total) in which they made an unspeeded choice as to which of three objects presented together was one of the six studied exemplars. In the first block, the objects were shown in the same viewpoint as during study, while they were in a new viewpoint for the second block. Participants were allowed to review the target exemplars shown together in a single display after trials 6 and 24 and instructed after trial 24 that the subsequent targets would differ in viewpoint. Participants completed the NOMT with three separate novel object categories: Greebles, Ziggerins, Sheinbugs (Fig. 1A). Performance was indexed by percent accuracy across all trials.

Fourth, participants completed the Vanderbilt Face Matching Test (VFMT; Sunday et al., 2015), which was designed to measure face recognition ability through a series of independent matching trials, using faces from both genders. On each trial, participants studied 2 faces, then were presented with a 3-alternative forced choice where they were instructed to indicate which face was studied. Matching faces varied from their original presentation in their position, perspective, and similarity to target. There were 96 total trials with performance indexed by percent accuracy across all trials. For a complete description of the VFMT, see Sunday et al. (2015).

Fifth, participants completed the Vanderbilt Car Matching Test (VCMT), which was modeled after the VFMT. On each trial, participants studied two cars, then were presented with a three-alternative forced choice where they were instructed to indicate which car was studied. There were 96 total trials, and performance was indexed by percent accuracy across all trials.

The sixth test was the Novel Object Matching Test (NOMaT), mirrored after the sequential matching tests with faces and cars. For the novel categories, however, the probe image size also varied randomly across trials, where half the trials presented the probe image in the same size as the study image (125 x 125 pixels) and half in a slightly smaller size (95 x 95 pixels). Participants completed 32 trials per novel object category (Ziggerin, Greeble, Sheinbug) for a total of 96 trials. There were equal numbers of same and different trials. Performance on this task was indexed by calculating sensitivity (d’).

Aggregate indices of face, car and novel object recognition were computed across standardized performance measures of the same categories. For each participant, an aggregate face index was calculated as the average standardized performance on the CFMT+ and VFMT. An aggregate car index was calculated as the average standardized performance on the CCMT and VCMT. Primary correlation analyses used the face and car aggregates as indices of face and car recognition ability, respectively. An aggregate novel object index was also calculated as the average standardized performance on the NOMT and NOMaT. In supplemental analyses, the novel object aggregate was included as a covariate for isolating domain-specific face and car effects, allowing us to remove individual variability explained by domain-general performance (e.g., McGugin et al., 2014).

### Whole-brain anatomic acquisition and analyses

Participants were scanned on a Philips 7-Tesla (7 T) Achieva MRI scanner with a quadrature transmit and 32-channel parallel receive array coil (Nova). For each participant, one imaging session was acquired, organized into three stages: 1) whole-brain anatomic imaging; 2) functional localization; and 3) ultra-high resolution susceptibility-weighted imaging. First, A 3D T1-weighted acquisition was acquired with the following parameters and used in real-time to identify structural landmarks for planning of subsequent ultra-high resolution scans: TR = 4.3 ms, TE = 1.90 ms (minimum), flip angle = 7°, TI = 1,300 ms, sagittal plane acquisition, FOV = 256 mm, 170 slices (200 slices for 30 subjects), voxel size = 1-mm isotropic. To compare the relative locations of functional activation peaks in AUT and TD groups, we normalized the T1-weighted anatomical scan in Montreal Neurological Institute (MNI) space (Fonov et al., 2009) using SPM12 (www.fil.ion.ucl.ac.uk/spm/).

We segmented our 3D T1-weighted acquisition to define an anatomical region of interest for each subject. Cortical reconstruction and segmentation of our whole-brain anatomical images were performed by using FreeSurfer (FS) v7.4.0 image analysis suite (Dale et al., 1999; Fischl & Dale, 2000). The automated FreeSurfer processing pipeline included motion correction, nonuniform intensity normalization for intensity inhomogeneity correction, removal of nonbrain tissue, transformation to Talairach space, and segmentation of the subcortical white matter and deep gray matter volumetric structures (Dale et al., 1999; Fischl et al., 2002). From the automatic segmentation in Freesurfer, we obtained a CT estimate of the right fusiform gyrus (Anatomical rFG) for each participant.

### Functional MRI acquisition, stimuli, design, and analyses

Each participant completed a functional localizer scan (160 dynamics/run). All functional scans were acquired using 3D PRESTO T2*-weighted imaging (TR/TE(shifted) = 29/35 ms, dyn. scan time = 2 s, flip angle = 12°, axial plane acquisition, FOV = 240 mm^2^, voxel dim. = 2.5 x 3.2 x 2.5 mm). We presented all images with MATLAB 2022 (www.mathworks.com) using Psychophysics Toolbox (Brainard, 1997). This localizer scan was used to identify the functional peak of face-selective areas in real time. We used grayscale images (36 faces, 36 objects) in a 1-back detection task across 20 alternating blocks of face and object images. Each block consisted of 16 trials in which a stimulus is presented for 900 ms followed by a 100 ms fixation. Stimuli included grayscale images of 35 faces, 35 objects, and 35 scrambled images. An exact image was repeated one to two times per block, and participants indicated with a button press when they saw the immediate repeat. Stimuli appeared randomly in three different sizes, and the same size never repeated in immediate succession. The run lasted 5.6 min.

#### Preprocessing

Functional MRI data were processed by using SPM12 and in-house MATLAB scripts. Functional data were first realigned and co-registered to the structural image by using a skull-stripped structural image as a source image. No spatial smoothing was applied. Functional data were co-registered to the ultra-high resolution mean image (see below), and analyses were performed in individual subject space. For the purpose of comparing peaks of functional activation across groups and with reports in the literature, we also normalized the functional data into MNI space and co-registered normalized functional maps to the normalized T1 weighted image.

#### Region-of-interest identification

Functional data analyses focused on the middle right face-selective patch in the fusiform gyrus, rFFA2. For each participant, a linear model was fit to the localizer run with a regressor for each domain (face and object) as well as six movement parameters (x-translation, y-translation, z-translation, pitch, roll, yaw). The linear model was then convolved with the standard HRF function, and a high-pass temporal filter of 128 s was applied to account for low frequency signal drift. Functional activation maps were generated using an uncorrected *p* <.001 and minimum cluster size of 5 voxels. We used the Marsbar toolbox (Brett et al., 2002) with a face > object contrast to define the rFFA2 as a sphere of 3-mm radius positioned on the peak of face selectivity on the right fusiform gyrus (Pinsk et al., 2009; Weiner et al., 2014b).

### Ultra-high resolution image acquisition and processing

Ultra-high resolution T2*-weighted images were acquired by using slice-selective gradient-echo acquisitions with real and imaginary images. We obtained a minimum of three ultra-high resolution acquisitions per participant (AUT: average = 5 scans; TD: average = 5 scans), with the following parameters: field of view = 240 x 180.194 x 21.9 mm, voxel resolution = 0.194 x 0.194 x 1.00 mm, 20 slices, 0.1-mm gap, “shortest” (878.8 ± 8.29 ms) repetition time, “shortest” (27.5 ± 0.31 ms) echo time, 27.26 pix water/fat shift, 55° flip angle, flow compensation, 9 min 11 ± 5.2 s total duration. From real and imaginary images, we calculated magnitude and phase images, which were then processed to create susceptibility weighted images (Haacke et al., 2004).

**Fig. 2:**
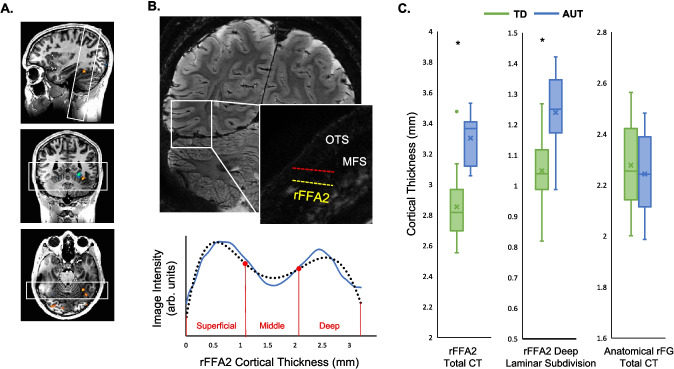
A. For a representative participant, functional data from the face localizer is overlaid on the anatomical T1-weighted scan. The right rFFA2 is identified as the contiguous cluster of voxels in the middle fusiform gyrus that is more activated by faces compared with common objects (hot colors are face-selective). White boxes show the strategic alignment of our ultrahigh-resolution slices perpendicular to the activated fusiform cortex (causing a rightward tilt to the ultrahigh-resolution coronal slices in B). **B.** Top: One ultrahigh-resolution slice is shown for a representative participant with autism. The inset zooms in on the right lateral fusiform gyrus, where this participant’s functionally defined rFFA2 falls between the occipital temporal sulcus (OTS) and the middle fusiform sulcus (MFS). The cortical boundaries of the rFFA2 are marked in yellow (superficial) and red (deep). Bottom: The line plot (blue line) represents the mean of all traces from the superficial border to the deep border (and vice versa) that fall between the borders of the rFFA2. A fourth order polynomial (black line) is fit to the mean. Points of inflection (red circles) denote changes in signal intensity and are used to isolate the middle layer from the superficial and deep laminar subdivisions. **C.** Box and whiskers plots show the means (x) and individual variability of thickness of the rFFA2 and its deep laminar subdivision, separated by group. The FFA was thicker in AUT compared with TD (*t* = 6.6, *p* <.001). This pattern was consistent in the deep (*t* = 6.12, *p* <.001) laminar subdivisions but not present in the anatomically defined right fusiform gyrus region of interest

Following McGugin et al. (2020), we planned the ultra-high resolution image acquisitions in real-time and on an individual basis, such that the frequency encoding direction aligned perpendicular to the individually-defined rFFA2 on the ventral surface of the temporal lobe. This careful alignment minimizes differences in partial volume effects across layers. Because of this alignment, ultra-high resolution slices viewed coronally will appeared tilted to the right (Fig. 2A-B) while the fusiform gyrus appears flat/horizontal.

Ultra-high resolution data were reconstructed at 0.1875 mm in-plane, and then scan acquisitions were co-registered to one another by using SPM12 (www.fil.ion.ucl.ac.uk/spm/) and averaged in MATLAB. The averaged image was loaded into GIMP, where trained image analysts manually traced the gray matter–white matter (deep) and gray matter–cerebrospinal fluid (superficial) cortical boundaries of the inferior temporal lobe of the right hemisphere. This method has shown very high interrater reliability: intraclass correlation = 0.98 (McGugin et al. 2020).

### FFA registration and trace identification

Each participant’s rFFA2 was co-registered to their average ultra-high resolution image and then overlaid as a mask on the manually segmented ultra-high resolution average image. The edges of the rFFA2 were traced for four to six slices depending on the individual subject’s representation, with measures taken to avoid distortion due to veins or image artifacts.

Using in-house MATLAB scripts (see McGugin et al., 2020, for details), we calculated the total regional CT as the distance from the superficial (gray matter–cerebrospinal fluid) border to the deep (gray matter–white matter) border for all slices. We then characterized the laminar structure as changes in signal intensity in the image. Traces were acquired as the series of lines originating at each superficial boundary voxel and terminating in each deep boundary voxel, and vice versa. Gray matter intensity was sampled across the length of the trace. A fourth-order polynomial was then fit to an average trace, with points of inflection defining the depths at which image intensity changed. Three laminar subdivisions were identified as the distances between (1) gray matter–cerebrospinal fluid border and the first inflection point (superficial layers, putatively supragranular layers I-III); (2) first and second inflection points (middle layer, putatively granular layer IV); and (3) second inflection point and the gray matter–white matter border (deep layers, putatively infragranular layers V–VI).

### Brain-behavior analysis

Within diagnostic groups, the correlation between face and car aggregate measures and cortical thickness was explored using Pearson’s correlation coefficient (*r*). The opposite correlation effect (OCE) for each group was calculated as the difference between the correlation of face aggregate x cortical thickness and car aggregate x cortical thickness. The standard error of the difference between OCEs was computed by using Fisher's transformation; then, a *t*-test was run to calculate the *t*-score and the two-tailed *p*-value for testing the hypothesis that the OCEs are different between groups. The same analyses was applied for rFFA2 total CT, rFFA2 deep layer CT, and rFG CT.

## Results

### Behavior

Group differences in behavior were significant only with faces, where TD outperformed AUT in the face matching test (CFMT: *t* = 2.7, *p* =.006, d =.93). Typical developmental histories also outperformed AUT in the face memory test (VFMT: *t* = 1.8, *p* =.04, d =.63, although this effect did not survive correction). No other group differences were significant (Fig. 1).

For subsequent analyses and as in McGugin et al., (2020), we aggregated the standardized performance across the tasks for each category. Estimating an ability for face, car, or object recognition based on an aggregate of two tasks allows us to emphasize the shared variance related to the category and deemphasize that associated with the specific task format. Going forward, we will use “Face,” “Car,” and “Novel Object” to represent the aggregate performance across memory and matching tests of like domains.

### Regions-of-interest

We defined two separate regions of interest for analyses. First, we defined our primary region based on face selectivity in our functional localizer (rFFA2) as in McGugin et al. (2020). Second, to test the benefits of our functional definition, we defined a second region anatomically over a larger portion of the right fusiform gyrus (anatomical rFG). While our specific hypotheses focused on the rFFA2, including the anatomical rFG allowed us to address the spatial selectivity of our results in rFFA2. We identified the rFFA2 in all participants using a face > object contrast and anatomical landmarks (i.e, the middle fusiform sulcus) as a guide to select the anterior-most cluster within the lateral FG: MNI x-, y-, z-coordinates (standard deviation); AUT: [40 (6), − 50 (7), − 17 (4)]; TD: [42 (5), − 53 (7), − 19 (6)]. For all subsequent analyses, we use the rFFA2 defined from the nonwarped and nonsmoothed functional data.

The right fusiform gyrus was defined as an anatomical region of interest in each participant, with mean volume of ~ 8,139 mm^3^ in TD and ~ 7,427 mm^3^ in AUT, so drastically larger than the 3 mm^3^ rFFA2 regions of interest.

### Total and laminar cortical thickness

The rFFA2 was thicker in individuals in the AUT group than the TD group (*t* = 6.57, *p* <.001, d = 2.3; Fig. 2B, left panel). The proportional representation of the laminar subdivisions relative to total regional CT was consistent with what would be expected from MR microscopy in area V4 in nonhuman primates, where each of the three subdivisions occupies roughly one-third of the cortical depth (Chen et al., 2012) for our AUT sample—superficial layers (~ 38%), middle layer (~ 30%), deep layers (~ 33%)—as well as the TD sample—superficial layers (~ 37%), middle layer (~ 34%), deep layers (~ 30%). However, mean thickness was greater in AUT at both the deep (*t* = 6.12, *p* <.001, d = 2.15) and superficial (*t* = 4.84, *p* <.001, d = 1.68) laminar subdivisions, whereas there was no group difference for the middle laminar subdivision (*t* =.28, *p* =.39, d =.09; Fig. 2B).

In contrast to Total CT in the functional rFFA2, Total CT in the anatomical rFG did not differ significantly across groups (*t* =.60, *p* =.28, d =.21; Fig. 2B, right panel).

### Does behavior predict regional and laminar CT?

**Fig. 3:**
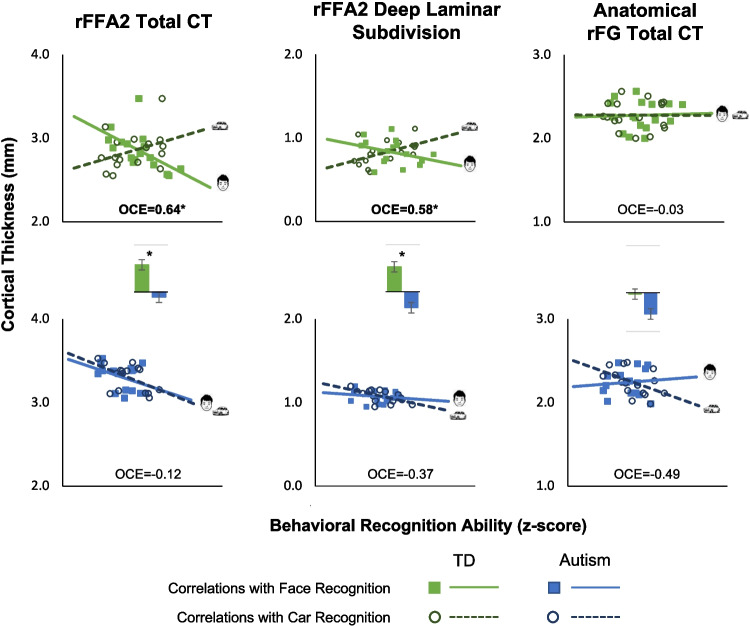
Scatterplots for typical development (TD; top row) and autism (bottom row) groups, showing correlations between face recognition ability (filled squares, solid line) or car recognition ability (hollow circles, dashed line) with cortical thickness. Correlations are shown for rFFA2 total CT (left panel), rFFA2 deep laminar subdivision (middle panel), and the anatomical rFG (right panel). The oppositive correlation effect (OCE) indices are bold if significant (*p* <.05). Bar graphs between scatterplots compare the OCE across groups. *Significant group difference (*p* <.05) was observed only for rFFA2 total CT and rFFA2 deep layers

Replicating previous work in our TD adults (Fig. 3), we found the signature opposite trend for face ability and car ability in their correlation with rFFA. Specifically, the OCE was significant for total CT of rFFA2 (*r* =.64, *p* =.006) and for CT of the deep laminar subdivision (*r* =.58, *p* =.015), with the pattern showing a negative relationship between Face recognition and CT, and a positive relationship between Car recognition and CT. In contrast, the OCE was not significant in our AUT group for total CT of rFFA2 (*r* =.12, *p* =.66), nor for CT of the deep laminar subdivision (*r* =.37, *p* =.16). We further tested whether the OCE was significantly different across groups and found that the OCE was significantly smaller in the AUT group than the TD group, in both total rFFA2 CT (z = − 2.28, *p* =.023) and in the deep laminar subdivision (z = − 2.73, *p* =.006). While our a priori hypotheses focused on total rFFA2 and the deep laminar subdivision, we report effect sizes for superficial and middle laminar subdivisions in Table 1, which may still be of interest for future efforts to conduct meta-analyses and extend upon the current results.

**Table 1:** Opposite correlation effect (OCE) for zero-order correlations with face and car recognition after controlling for novel object recognition

	rFFA2 total CT	rFFA2 laminar subdivisions			Anatomical rFG
		Deep	Middle	Superficial	
**Typical development**	0.64	0.58	0.24	0.30	− 0.03
**Autism**	− 0.12	− 0.37	− 0.14	0.22	− 0.49

Finally, we report the OCE as an effect size in the anatomical rFG (Fig. 3, right panel). Illustrating that the pattern we are interested in is specific to the functional region of interest for the TD group, the pattern in the rFFA2 was different from that in the rFG (William’s test for dependent correlations: *t* = 1.87, *p* =.08). Specifically, in TD, the OCE was not significant in rFG (*r* =.03, *p* =.91). In AUT, the OCE effect size was high (*r* =.49, *p* =.05), but intriguingly, the pattern was opposite to what we observe at the level of rFFA2 in TD. Also, it is worth noting that the OCE was not significantly different between groups (z = − 1.31, *p* =.19).

We also examined effects with novel objects (see Supplement). Recognition ability for novel objects did not predict cortical thickness at any scale for either group, consistent with the hypothesis that these effects in rFFA2 are being driven by experience with familiar categories, as opposed to general object recognition ability. Category-specific effects after regressing out the influence of novel objects are qualitatively the same.

## Discussion

We found, as others have previously, that face recognition is more affected in autism than nonface object recognition. Using an individual differences approach, we explored the association between these behavioral deficits and the structure of the rFFA2 between two groups: individual with autism and neurotypical controls. It is a challenge to compare patterns of correlations between behavior and brain structure between two groups with sufficient statistical power. For instance, even without comparing groups, a study (Meyer et al., 2019) analyzing data from 854 participants from the Human Connectome Project (Van Essen et al., 2013) failed to find a specific link between CT in face-selective areas and face recognition. Our approach offered two advantages towards tackling the question of power. First, we measured face and car recognition abilities with tasks that have strong psychometric properties and averaged two different tasks in each case to produce more robust estimates. Second, the pattern of interest, the crossover interaction between car and face recognition in their relationship to CT, provided an unusually large effect size. The independence of the two opposite correlations in (McGugin et al., 2020) allowed us to simplify our predictions by using the OCE and, thereby, increase our statistical power. We multiplied face recognition ability by − 1 and summed it with car recognition ability, before correlating it with CT to create the OCE. The OCE varied from − 1 to 1—was maximal when the two correlations were large and in opposite directions and minimal when both correlations were weak or when both types of recognition abilities show the same patterns of association with CT (thereby cancelling each other). Using the OCE, we gain power by foregoing the ability to test each pattern separately. The effect size (a critical determinant of power) for the OCE was larger than each of the correlations on their own.

With ultra-high resolution imaging, a functional region of interest approach and measures of ability with solid psychometric properties, we replicated the finding of a large OCE in neurotypical adults from McGugin et al. (2020), both in total rFFA2 CT and in the deep laminar subdivision. The OCE was large in this group, because (as in McGugin et al., 2020) those with better face recognition had relatively thinner cortex, whereas those with better car recognition had relatively thicker cortex. Critically, our autistic group showed a different pattern. The OCE was absent in this group, indeed numerically reversed, because those with better face and car recognition had thinner cortex, and the effect was numerically larger for cars. The significant interaction whereby the OCE in rFFA2 is reduced in adults with autism is consistent with the prediction that the root of this effect, previously uncovered in TD adults, lies in development.

What mechanisms drive this difference between groups is not revealed by our results. The account proposed by McGugin et al. (2020) is that the OCE is observed in neurotypical adults, because they learn about faces early in life, shaping the rFFA2 in ways that are unique to early brain development (which could include greater pruning of deep layers and/or greater myelination adjacent to deep layers). A thinner cortex as measured with MRI, specifically a thinner deep layer subdivision, may result from differences in white matter tracts connecting the FFA to other areas, and indeed, the integrity of white matter tracts from FFA to the anterior temporal lobe also predicts face recognition ability (Gomez et al., 2015). Relative to neurotypical adults, autistic individuals show hypoconnectivity and atypical development of functional connectivity between core and extended face network regions (Lynn et al., 2018), which is in line with the overall thicker cortex that we observe in our sample. In contrast to face expertise, any car expertise would come relatively later in time, when other neural mechanisms are in play. There are reasons to believe that face recognition and car recognition are acquired closer in time in autistic individuals, which led us to predict a reduced OCE. Infants and toddlers who develop autism show elevated attention to objects (Ibanez et al., 2008), diminished attention to faces (Bhat et al., 2010; Jones et al., 2016), and better working memory for objects than faces (Noland et al., 2010), relative to low-likelihood comparison infants. Furthermore, early preference for nonsocial vs. social stimuli is associated with later symptom severity (Bacon et al., 2020). Preschoolers with autism show preferential attention to nonsocial objects (Wang et al., 2020), and evoked encephalographic responses to faces is selectively impaired in autism while it is intact to objects (Dawson et al., 2002). The evidence is mixed as to how this phenotype relates to clinical course.

There is considerable variability among infants in attentional preference for faces compared with objects, with nearly half that variability attributable to genetic factors (Portugal et al., 2024), and fairly limited evidence for association with genetic risk for autism or later autism symptoms (Falck-Ytter, 2024). Critically, while we observed a reduced OCE in autistic individuals, the use of OCE (necessary to achieve power) masks the specific pattern of result observed in this group. That is, an absent OCE could have resulted from a negative correlation with CT for both cars and faces, or a positive correlation with CT for both cars and faces. This last result might have been predicted on the basis that face expertise in autistic individuals may have been delayed and thus closer in developmental time to that for cars. Contrary to this expectation, the effects for both faces and cars in the AUT group were more similar to those for faces in the TD group. This could be consistent with reports of reduced attenuation to both social and nonsocial stimuli in habituation paradigms administered to infants at elevated likelihood of autism (Hendry et al., 2018). Other work also reports “sticky attention” in high likelihood infants (Sacrey et al., 2013), which may result in parallel increases in attention to both social and nonsocial stimuli. Recently, it has been shown that autistic individuals, compared with neurotypical controls, have impoverished visual experience with faces, including reduced exposure duration, increased viewing distances, and biases toward nonfrontal poses (Kamensek et al., 2024). Future work in developmental samples could distinguish between these explanations.

What is perhaps most telling in our results is that the OCE in TD, and the difference between groups, is local to the rFFA2. We found no evidence that individual differences in CT in the surrounding FG cortex are related to face or car recognition abilities. This suggests the group difference is owing to local specialization in the rFFA2 in the TD group, which had not occurred in the AUT group. Future work should explore an expanded field of view in humans to investigate whether the effect observed in AUT in the rFG is being carried by another functional region (e.g., the more posterior FFA1). Indeed, a recent study reported unique histo-architectonic features (e.g., cytochrome oxidase and myelin) of a middle face patch compared with surrounding face-selective patches in nonhuman primates (Oishi et al., 2024), suggesting the functional specialization of the middle face patch has associated structural correlates. Critically, the quantitative architectonic distinctions observed in the middle face patch relative to other inferotemporal face patches was most pronounced in the superficial layers. While we did not have a priori hypotheses for effects in superficial layers, architectonic variability across layers, regions, and populations will have important implications regarding the interconnectivity of face processing.

We also found that the rFFA2 was thicker overall in adults with AUT compared with neurotypical adults. This is in contrast to findings of cortical thinning in anatomically defined temporal regions in adolescents and adults with AUT (Wallace et al., 2010) and the proposed pattern of brief early overgrowth followed by extended accelerated thinning (Zielinski et al., 2014) that can result in thinner cortex when measured cross-sectionally in autistic adults. A meta-analysis also reported reduced CT of the fusiform gyrus as a whole in AUT (Patriquin et al., 2016). Importantly, these prior studies have not performed structural measurement on functionally defined rFFA2 s on an individual-by-individual basis, and their results should more appropriately be compared to our results in the anatomical rFG, where we did not find a significant difference between groups. The fusiform gyrus encompasses multiple functional regions (Chen et al., 2023), so using a group template, probabilistic atlas or anatomical atlas will have an inherently underestimated effect size (Oishi et al., 2024). A plausible reason for differences in local versus global CT between groups is that early specialization for faces drove the relative thinning of the rFFA2 in neurotypical individuals, by the same logic that those within the neurotypical group who have better face recognition may have thinner rFFA2.

In a similar vein, recent work suggests that variability of cortical thickness of the middle fusiform sulcus is greater in AUT relative to TD (Ammons et al., 2021; Ramos Benitez et al., 2024), but within our small functionally defined region, we did not replicate the finding of greater variability in AUT. Specifically, we find no significant group difference in total FFA CT variance (F = 1.18, *p* =.755, η^2^ =.034). We do find a group effect in variance at the deep laminar level, but in the opposite direction as that reported by previous work: variance in deep layer thickness is greater in TD than AUT (F = 5.37, *p* =.002, η^2^ =.148). Future work should explore how individual differences in face or object recognition may interact with group variance effects.

## Limitations and future directions

Our study has number of strengths, including ultrahigh-resolution imaging affording laminar analyses, a comprehensive and rigorous behavioral battery, an individual differences approach, and a structural approach that allowed us to avoid a number of common confounds in the study of face processing in autism. There are some notable limitations; for example, our conclusions about the developmental trajectory of cortical development as it relates to visual experience are speculative.

In addition to the opportunities introduced above, future work should measure the structural correlates of visual experience during early development to expand upon the results found in adults. Future work should also explore the role gender plays in behavior x cortical thickness associations. We did not have enough power with the current sample size to specifically assess sex or gender differences, but future work should set out to do so. In particular, women tend to outperform men in face recognition (Herlitz & Lovén, 2013), whereas more men are interested in cars, resulting in an advantage in performance (McGugin et al., 2012). Given the higher ratio of men to women (roughly 4:1) in autism, focused efforts should be made to recruit women to comprehensively address gender effects. In addition, it would be ideal to find a category other than cars that produces an OCE with faces but is of greater interest to women or, even better, gender neutral.

## Conclusions

We explored the relationship between gray matter CT of the FFA and face recognition ability in adults with and without autism. Autistic individuals have an altered development of face recognition skills; as such, we predicted a unique relationship between FFA CT and face recognition in autism. Our results replicated the OCE in total FFA CT and in deep layers in neurotypical adults where individuals with better face recognition had relatively thinner FFAs, while those with better car recognition had thicker FFAs. Importantly, we find a significant reduction of this OCE in autistic adults. We demonstrate that the abnormal OCE in autism is specific to the right FFA2. The current study offers insight into the neural basis of face recognition deficits in autism, as well as insight into the developmental role of object recognition in neurotypical adults.

## Supplementary information

Below is the link to the electronic supplementary material.

Supplementary file1 (DOCX 197 KB)

## Data Availability

All data supporting the findings of this study are available upon reasonable request to the corresponding author, R.M.

## Code availability

All code used in this study are available upon reasonable request to the corresponding author, R.M.
