# Supplementary material for: Ultra-high resolution imaging of laminar thickness in face-selective cortex in autism
Source: Cogn Affect Behav Neurosci. 2025 Apr 30;25(4):1210–23. doi: 10.3758/s13415-025-01298-w (PMC12356732; doi:10.3758/s13415-025-01298-w)
Supplement: Supplementary file 1 — Supplementary file1 (DOCX 197 KB) [file 13415_2025_1298_MOESM1_ESM.docx]

**Ultra-high resolution imaging of laminar thickness in face-selective cortex in autism**

**SUPPLEMENTAL SECTION**

**Supplemental Figure 1**. Scatterplots for Typical Development (TD; top row) and Autism (bottom row) groups, showing correlations between face recognition ability controlling for performance on novel objects (filled squares, solid line) or car recognition ability controlling for performance on novel objects (hollow circles, dashed line) with cortical thickness. Correlations are shown for rFFA2 total CT (left panel), rFFA2 deep laminar subdivision (middle panel), and the anatomical rFG (right panel). The oppositive correlation effect (OCE) indices are bold if significant (p<.05). Bar graphs between scatterplots compare the OCE across groups. Asterisks represent a significant group difference (p<.05), which was observed only for rFFA2 Total CT and rFFA2 Deep Layers.

|  | **rFFA2 Total CT** | **rFFA2 Laminar Subdivisions** | | | **Anatomical rFG** |
| --- | --- | --- | --- | --- | --- |
|  |  | **Deep** | **Middle** | **Superficial** |  |
| **Typical Development** | 0.60 | 0.58 | 0.22 | 0.25 | 0.02 |
| **Autism** | -0.12 | -0.37 | -0.14 | 0.23 | -0.44 |

**Supplemental Table 1**. Opposite Correlation Effect (OCEs), for partial correlations with Face and Car Recognition controlling for Novel Object Recognition
